# Mertk on tumor macrophages is a therapeutic target to prevent tumor recurrence following radiation therapy

**DOI:** 10.18632/oncotarget.11823

**Published:** 2016-09-02

**Authors:** Marka R. Crittenden, Jason Baird, David Friedman, Talicia Savage, Lauren Uhde, Alejandro Alice, Benjamin Cottam, Kristina Young, Pippa Newell, Cynthia Nguyen, Shelly Bambina, Gwen Kramer, Emmanuel Akporiaye, Anna Malecka, Andrew Jackson, Michael J. Gough

**Affiliations:** ^1^ Earle A. Chiles Research Institute, Robert W. Franz Cancer Center, Providence Portland Medical Center, Portland OR, USA; ^2^ The Oregon Clinic, Portland OR, USA; ^3^ Providence Hepatobiliary and Pancreatic Cancer Program, Providence Portland Medical Center, Portland OR, USA; ^4^ Host-Tumour Interactions Group, Division of Cancer and Stem Cells, University of Nottingham, UK

**Keywords:** radiation, macrophage, tumor, phagocytosis, apoptosis

## Abstract

Radiation therapy provides a means to kill large numbers of cancer cells in a controlled location resulting in the release of tumor-specific antigens and endogenous adjuvants. However, by activating pathways involved in apoptotic cell recognition and phagocytosis, irradiated cancer cells engender suppressive phenotypes in macrophages. We demonstrate that the macrophage-specific phagocytic receptor, Mertk is upregulated in macrophages in the tumor following radiation therapy. Ligation of Mertk on macrophages results in anti-inflammatory cytokine responses via NF-kB p50 upregulation, which in turn limits tumor control following radiation therapy. We demonstrate that in immunogenic tumors, loss of Mertk is sufficient to permit tumor cure following radiation therapy. However, in poorly immunogenic tumors, TGFb inhibition is also required to result in tumor cure following radiation therapy. These data demonstrate that Mertk is a highly specific target whose absence permits tumor control in combination with radiation therapy.

## INTRODUCTION

High-dose radiation has the ability to produce large-scale cancer cell death at the tumor site over a short time frame [1]. Cancer cell death has the potential to provide both antigen and the immunological adjuvants required for effective adaptive immune responses [2]. For these reasons, it has been proposed that killing cancer cells in a way that releases both antigen and adjuvant, so called immunogenic cell death, could generate more effective adaptive immune responses to tumor antigens. For this immune priming effect to be fully functional, it is critical to consider both the way that cancer cells die [3], the mix of immune cells in the vicinity of dying cancer cells [4, 5], and it is just as critical to consider the impact of radiation directly on the immune cells. This is particularly relevant since cell-associated antigen and adjuvant are commonly released from many tumors: necrotic foci are frequently observable in advanced cancers [6], and uric acid, which has been shown to act as an immune adjuvant [7], is frequently elevated in the blood of cancer patients receiving cytotoxic therapy. These data would suggest that tumor antigen-specific immune responses should be efficiently generated by most large tumors with necrotic foci without additional treatment, or each time cytotoxic therapy is delivered. However, evidence suggests that adaptive immune responses to tumors are not constantly growing in strength, by contrast in the absence of additional intervention, adaptive immune responses to tumors may be present but are generally ineffective in tumor control [8–11].

To understand this disconnect, it is important to appreciate that there are simultaneous negative signals that influence the response to adjuvant and thereby limit adaptive immune responses against tumor antigens *in vivo*. As tumors progress in mice and in patients there is a progressive macrophage infiltration that has been shown to be critical for tumor angiogenesis, progression and metastatic spread in mice [12, 13] and is associated with poor prognosis in patients [14]. There is often a reciprocal relationship between T cell infiltration and macrophage infiltration in tumors, and where T cells are low and macrophages are high, the prognosis is especially poor [15, 16]. Tumor macrophages are generally differentiated into phenotypes that can suppress T cell activation [17], and treatment of these tumor macrophages with immunological adjuvants results in production of anti-inflammatory rather than pro-inflammatory cytokines [18]. In addition to this pre-disposition of tumor macrophages, dying cells are efficiently immunosuppressive *in vitro* and *in vivo*. *In vitro*, apoptotic cells drive differentiation of macrophages into suppressive phenotypes that involve secretion of anti-inflammatory cytokines such as TGFβ and IL-10 and upregulation of suppressive molecules such as Arginase I [19–21]. *In vivo*, systemic administration of apoptotic cells is an efficient means to generate antigen-specific tolerance [22, 23].

Mertk, along with Tyro3 and Axl, are members of the TAM (Tyro3-Axl-Mertk) subgroup of receptor tyrosine kinases. The dominant ligand for Mertk is Gas6, a protein S-related gene that has been shown to bind exposed phosphatidylserine (PS) on apoptotic cells. The Mertk-Gas6-PS interaction results in phagocytosis of the apoptotic cell [24], and mice with inactivated Mertk are defective in their handling of apoptotic cells [25, 26]. Complete loss of Mertk and its family members Akt and Tyro3 results in pro-inflammatory patterns of gene expression and loss of immune privilege [27, 28]. Additionally, mice with inactivated Mertk are also hypersensitive to LPS stimulation [29], suggesting that in the steady state, Mertk delivers a negative regulatory signal. Mertk-dependent uptake of dying cells results in signal transduction that suppresses macrophage inflammatory responses resulting in strong immune tolerance to antigens that are taken up via Mertk ligation [30]. A number of other bridging molecules have been identified that have the capacity to link apoptotic cells to macrophages (reviewed in [31]). The relative contribution of the different adaptor molecules remains to be determined since where large-scale death occurs disruption of any one of these interactions may be sufficient to alter the handling of dying cells. However, unlike many other characterized receptors Mertk has the capacity to transduce signals to the macrophage and is the signaling component of phagocytic complexes involving MFGE8 [32] and C1q [33]. In some circumstances, these positive signals may be countered by phosphatases activated by CD47 ligation of SIRPa to block phagocytosis (reviewed in [34]). Thus, while a range of molecules may be involved in macrophage binding of opsonized apoptotic cells, there is evidence that Mertk in macrophages may play a central role in signal transduction following binding.

We and others have demonstrated increased infiltration of macrophages into tumors following radiation therapy [18, 35–38], and that these macrophages exhibited increased suppressive differentiation in the post-treatment tumor environment [18]. In studying the gene expression patterns of tumor infiltrating macrophages, we identified upregulation of Mertk in tumor macrophages following radiation therapy. We demonstrate here that ligation of Mertk on macrophages recapitulates the suppressive effect of dying cells and that blocking Mertk interaction with irradiated cancer cells blocks suppressive differentiation of tumor macrophages. Using CT-guided radiation to treat tumors in immune competent mice, we demonstrate that in immunogenic tumors, radiation therapy is curative in mice lacking Mertk. In poorly immunogenic tumors, loss of Mertk is not sufficient to change the response to radiation therapy, but when combined with blockade of TGFβR results in tumor cures. These data demonstrate that Mertk is a strong therapeutic target to permit tumor clearance by radiation therapy.

## RESULTS

**Figure 1:**
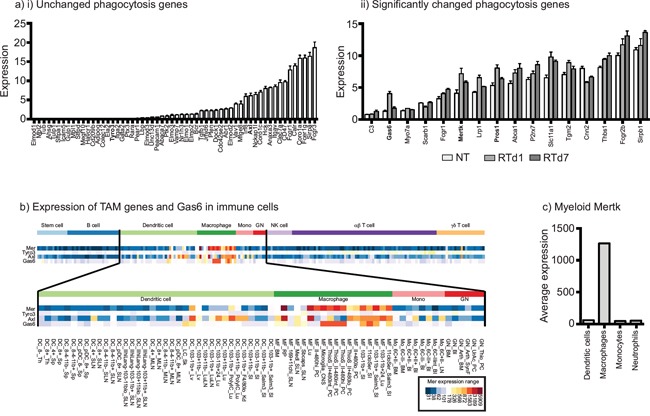
Upregulation of the macrophage-specific phagocytic receptor Mertk following radiation therapy of tumors **a.** CD11b^+^Gr1^lo^IA^+^ macrophages sorted from untreated and irradiated tumors were subjected to gene expression analysis (GEO accession number GSE34206). i) Phagocytosis-related genes were separated into those with significant changes in red, and those with no change following radiation (blue). ii) Graph shows normalized gene expression of individual phagocytosis-related genes that significantly change following radiation therapy, sorted by initial expression level. **b.** Expression of Mertk, Tyro3 Axl and Gas6 in murine immune cell populations analyzed by the Immunological Genome Project Consortium. **c.** Average expression of Mertk from the datasets shown in b), grouped by defined dendritic cell, macrophage, monocyte and neutrophil subsets.

A number of investigators have demonstrated that macrophages are recruited to tumors following radiation therapy and limit tumor control [18, 35–38]. These macrophages are polarized to M2 phenotypes determined by differentiation marker expression and gene array analysis of CD11b^+^Gr1^lo^IA^+^ macrophages sorted from untreated and irradiated tumors [18]. Since phagocytosis of dying cells is known to drive M2 differentiation of macrophages [19–21], we analyzed our dataset for genes involved in phagocytosis pathways that are significantly changed following radiation therapy (Figure 1ai-ii). Amongst this set of genes, it was noticeable that the phagocytic receptor for apoptotic cells Mertk was significantly upregulated early following radiation therapy, as was its ligands Gas6 and protein S (Pros1) (Figure 1aii). As a member of the TAM family of receptor tyrosine kinases, Mertk is known to play a significant role in the phagocytosis of dying cells. Interestingly, Mertk has been described as one of the most specific genes to distinguish macrophages from dendritic cells [39]. Comparative analysis of the TAM family members and Gas6 in the broad panel of immune cells made available through the Immunological Genome Project Consortium (www.immgen.org) shows that Mertk is highly restricted to macrophages, while Axl has slightly wider expression and Tyro3 is poorly expressed (Figure 1b). By grouping macrophage and other myeloid subtypes within the Immunological Genome Project Consortium dataset, it is clear that Mertk expression is highly restricted to macrophages, with above 20 fold higher expression on collected macrophage subtypes than dendritic cell subtypes, neutrophil subtypes or monocyte subtypes (Figure 1c). These data suggest that Mertk is a strong candidate as a target molecule on macrophages involved in responses to dying cells in the irradiated tumor.

**Figure 2:**
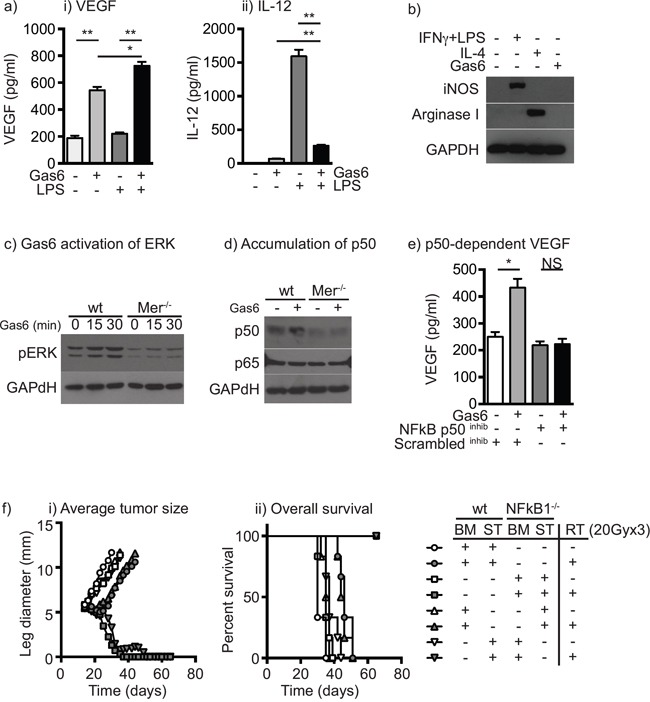
Ligation of Mertk results in a switch to suppressive cytokine patterns via upregulation of NF-kB p50 **a.** Bone marrow macrophages were left untreated or treated with Gas6 for 24 hours then left untreated or stimulated with LPS for a further 24 hours. Secretion of i) VEGF and ii) IL-12 was determined by cytokine bead assay. **b.** Bone marrow macrophages were left untreated, treated with IFNγ+LPS to drive M1 differentiation, IL-4 to drive M2 differentiation, or Gas6. Lysates were prepared after 24 hours and analyzed for expression of iNOS, Arginase I and GAPDH loading control. **c.** Bone marrow macrophages from wild-type or Mertk^−/−^ mice were left untreated or treated with Gas6 for 15 or 30 minutes, lysed and analyzed for phospho-ERK or GAPDH loading control by western blotting. **d.** Bone marrow macrophages from wild-type or Mertk^−/−^ mice were left untreated or treated with Gas6 for 24 hours, lysed and analyzed for NF-kB p50, NF-kB p65 or GAPDH loading control by western blotting. **e.** Bone marrow macrophages were left untreated or treated with Gas6 for 24 hours then treated with an NF-kB p50 inhibitor peptide or scrambled control peptide and stimulated with LPS for a further 24 hours. Secretion of VEGF was determined by cytokine bead assay. **f.** Wild-type or NFKB1^−/−^ mice were irradiated with 9.5Gy total body radiation and reconstituted with bone marrow from wild-type (wt) or NFKB1^−/−^ mice to create four types of bone marrow chimeras: wt bone marrow (BM) and wt stroma (ST) – *circles*; NFKB1^−/−^ BM and NFKB1^−/−^ ST – *squares*; wt BM and NFKB1^−/−^ ST – *upward triangles*; or NFKB1^−/−^ BM and wt ST – *downward triangles*. Following reconstitution mice were challenged with Panc02 tumors and at d14 left untreated (*open shapes*) or treated with 20Gy x3 focal RT (*filled shapes*). Graphs show i) average tumor diameter or ii) overall survival. Results are representative of two or more experimental repeats of 6-8 mice per group. Key: *=p<0.05; **=p<0.01; NS = not significant.

To understand how the phagocytic receptor Mertk may cause changes in macrophages, we examined the response to Mertk ligation *in vitro*. Bone marrow macrophages generated through culture in M-CSF were confirmed to express Mertk by immunofluorescence (data not shown). Treatment with Gas6 caused VEGF secretion in bone marrow macrophages (Figure 2ai) and suppressed the ability of macrophages to secrete TNFα (not shown) and IL-12 in response to LPS stimulation (Figure 2aii). To determine whether Gas6 could cause differentiation of macrophages into M1 or M2 patterns of gene expression, bone marrow macrophages were treated with Gas6 for 24 hours alongside controls treated with IFNγ and LPS to drive M1 differentiation, or IL-4 to drive M2 differentiation. Gas6 was unable to drive induction of the M1 marker iNOS or the M2 marker Arginase (Figure 2b). These data demonstrate that Mertk ligation affects cytokine responses without affecting expression of the prototypical markers of M1 and M2 differentiated cells.

Macrophages have been shown to change their cytokine response to stimulation through a change in the level of NFκB p50 in cells, as a result of signaling through NFκB p50 homodimers rather than conventional NFκB p50:p65 heterodimers [40] and we have demonstrated that NFκB1^−/−^ macrophages do not change their cytokine response in the presence of irradiated cancer cells [18]. Thus, alteration in NFκB p50 is a potential signaling mechanism to repolarize cytokine responses without changing differentiation. To confirm that Mertk signaling results in activation of the MEK-ERK-Tpl2- NFκB p105 complex, bone-marrow macrophages from wild-type or Mertk^−/−^ mice were treated with Gas6 and first analyzed for Erk phosphorylation. Gas6 increased Erk phosphorylation in wild-type, but not Mertk^−/−^ macrophages (Figure 2c), and 24 hours of treatment with Gas6 resulted in accumulation of NFκB p50 in wild-type but not Mertk^−/−^ macrophages (Figure 2d). To determine whether the cytokine response was due to the effects of NFκB p50, wild-type macrophages were treated with a cell-permeable NFκB p50 inhibitor peptide or a control peptide, and treated with Gas6. The presence of the NFκB p50 inhibitor significantly reduced VEGF induction (Figure 2e) while simultaneously increasing TNFα production (not shown) consistent with our prior studies using NFκB1^−/−^ macrophages [18]. In addition, Gas6 treatment did not induce VEGF in Mertk^−/−^ macrophages (not shown). To examine the role of myeloid expression of NFκB p50 in the response to radiation therapy, we established bone marrow chimeras where wild-type mice were given bone marrow from wild-type controls or NFκB1^−/−^ mice, and NFκB1^−/−^ mice were given bone marrow from NFκB1^−/−^ controls or wild-type mice. Once reconstituted, mice were challenged with Panc02 pancreatic adenocarcinoma tumors and then treated with radiation therapy (Figure 2f). As expected, radiation therapy causes a transient growth delay in wild-type mice, but in NFκB1^−/−^ mice radiation therapy results in tumor cures [18]. Importantly, this effect was entirely dependent on bone-marrow derived cells, such that wild-type mice with NFκB1^−/−^ bone marrow were cured by radiation therapy, while NFκB1^−/−^ mice with wild-type bone marrow exhibited only a growth delay (Figure 2f). These data demonstrate that activation of Mertk alters the cytokine response of macrophages via accumulation of NFκB p50, and that preventing NFκB p50 accumulation in hematopoietic cells through NFκB1^−/−^ dramatically improves the response to radiation therapy.

**Figure 3:**
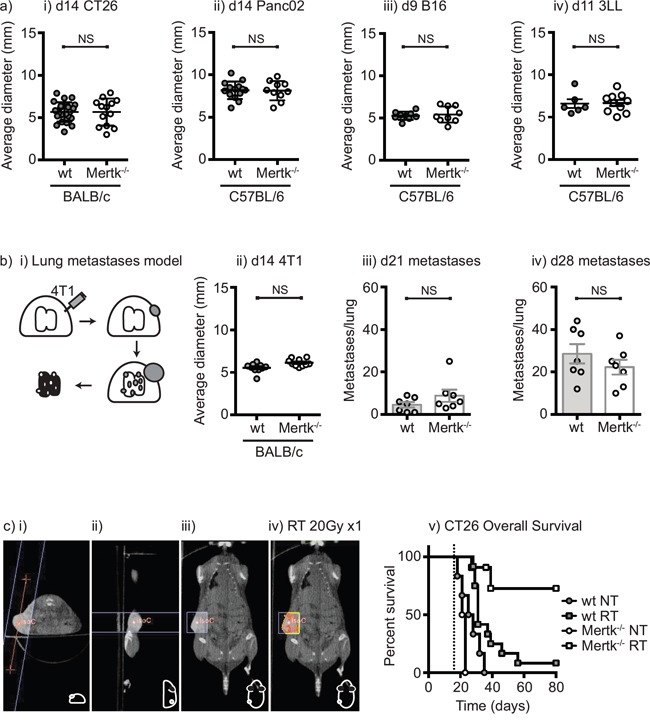
Tumor growth, metastatic spread and response to treatment in Mertk^−/−^ mice **a.** i) BALB/c wild-type or BALB/c Mertk^−/−^ mice were challenged with CT26 colorectal carcinoma, or C57BL/6 wild-type or C57BL/6 Mertk^−/−^ mice were challenged with ii) Panc02 pancreatic adenocarcinoma, iii) B16 melanoma or iv) 3LL lung adenocarcinoma. Graphs show average tumor diameters, where each symbol represents one mouse. **b.** i) BALB/c wild-type or BALB/c Mertk^−/−^ mice were challenged with orthotopic 4T1 breast carcinoma. Graphs show ii) average tumor diameter at d14, and lung metastases counts at iii) day 21 and iv) day 28, where each symbol represents one mouse. **c.** BALB/c wild-type or BALB/c Mertk^−/−^ mice were challenged with CT26 colorectal carcinoma and tumors were left untreated (NT) or treated on d14 with 20Gy x1 of focal radiation to the tumor (RT). i)-iii) CT imaging of tumor-bearing mice with isocenter marked as a red dot. iv) Dosimetry of radiation delivery to the tumor. Line represents boundary of beam. v) Graph shows overall survival. Results are representative of two or more experimental repeats of 6-8 mice per group. Key: NS = not significant.

Chimeric mice established with Mertk^−/−^ bone marrow have been shown to have delayed tumor formation compared to mice with wild-type bone marrow [41]. This was associated with early inflammation in the tumor implantation site of mice with Mertk^−/−^ bone marrow resulting in a CD8 T cell suppression of tumor growth [41]. To determine whether the absence of Mertk affects tumor growth or the response to radiation therapy, we established CT26 colorectal carcinoma in wild-type BALB/c or BALB/c Mertk^−/−^ mice and Panc02 pancreatic adenocarcinoma, 3LL lung adenocarcinomas, and B16 melanoma in wild-type C57BL/6 or C57BL/6 Mertk^−/−^ mice. Tumor growth was unaffected in Mertk^−/−^ mice on either background with any of the cell lines tested (Figure 3a). To determine the effect of Mertk on metastases, we used the 4T1 mammary carcinoma model in BALB/c mice, which has a high rate of spontaneous metastases (Figure 3b). We found that as in the other models, tumor growth was not affected in Mertk^−/−^ mice and the number of lung metastases was also not affected in mice lacking Mertk (Figure 3b). These data demonstrate that in a wide range of models in two different genetic backgrounds in our laboratory, the absence of Mertk does not affect tumor growth, progression or spontaneous metastases. To deliver a high rate of cell death into these tumors, CT26 tumor-bearing mice were treated with CT-guided radiation therapy to deliver therapeutic doses of radiation to the tumor while avoiding radiosensitive tissues (Figure 3c). As expected, radiation therapy resulted in transient control of CT26 tumors in wild-type mice followed by recurrence in 11 of 12 animals. However, in Mertk^−/−^ mice radiation therapy resulted in complete tumor regression in 9 of 11 animals (Figure 3c). These data demonstrate that the loss of Mertk results in significantly enhanced survival of mice following radiation therapy. This does not occur in untreated mice, but occurs as a result of complete tumor cure after treatment of Mertk^−/−^ mice with radiation therapy as compared to transient tumor control followed by outgrowth in wild-type mice.

**Figure 4:**
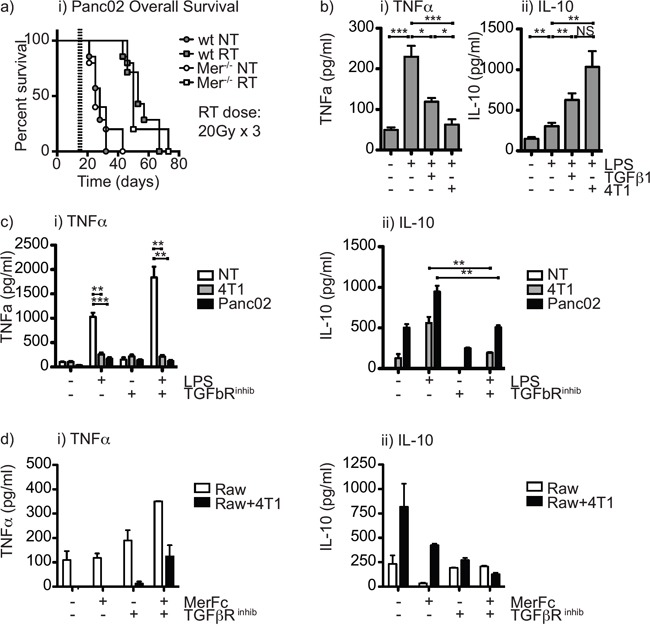
The combination of Mertk knockout and TGFβ inhibition restores proinflammatory function of macrophages in the presence of irradiated cancer cells **a.** C57BL/6 wild-type or C57BL/6 Mertk^−/−^ mice were challenged with Panc02 pancreatic adenocarcinoma and tumors were left untreated (*circles*) or treated on d14 with 20Gy x3 of focal radiation to the tumor (*squares*). Graph shows overall survival. **b.** Raw264.7 macrophages were left untreated or treated with TGFβ or co-culture with cancer cells irradiated at a dose of 10Gy, and incubated for 24 hours, then treated with 100ng/ml LPS and supernatant collected after a further 48 hours. Graphs show secretion of i) TNFα and ii) IL-10. **c.** Raw264.7 macrophages were left untreated or treated with irradiated (10Gy) cancer cells for 24 hours in the presence or absence of TGFβR inhibitor, then treated with 100ng/ml LPS and supernatant collected after a further 48 hours. Graphs show secretion of i) TNFα and ii) IL-10. **d.** Raw264.7 macrophages were left untreated or treated with irradiated (10Gy) cancer cells for 24 hours in the presence or absence of TGFβR inhibitor and MerFc blocking reagent, then treated with 100ng/ml LPS and supernatant collected after a further 48 hours. Graphs show secretion of i) TNFα and ii) IL-10. Results are representative of two or more experimental repeats. Key: *=p<0.05; **=p<0.01; ***=p<0.001; NS = not significant.

Surprisingly, in the Panc02 pancreatic adenocarcinoma model the effect of radiation therapy was not significantly different in Mertk^−/−^ mice from wild-type mice (Figure 4a). We have seen similarly divergent results in prior studies with CT26 and Panc02: CT26 tumors were highly responsive to TGFβR blockade in combination with radiation therapy, while Panc02 tumors remained poorly responsive to radiation therapy despite an improved immune environment following TGFβR blockade [42]. Exposure to TGFβ diverts macrophage pro-inflammatory responses towards M2-type responses [43, 44]; however, in in the CT26 tumor model, TGFβR blockade appeared to function mainly by improving CD8 T cell control of tumors [42]. We hypothesized that in the poorly responsive Panc02 pancreatic adenocarcinomas, both Mertk and TGFβ were acting together to suppress local immune responses by driving suppressive differentiation of tumor macrophages. To test whether Mertk and TGFβ act in combination, we made use of Raw264.7 macrophages that strongly repolarize from a constitutive M1 differentiation and cytokine pattern to M2 differentiation and cytokine pattern on exposure to irradiated cancer cells [18]. Treatment of Raw264.7 macrophages with TGFβ was also able to divert to IL-10 production rather than TNFα following LPS stimulation (Figure 4b), confirming the suppressive effect of TGFβ in this *in vitro* model. In our prior studies, treatment of macrophages with TGFβ did not affect expression of iNOS or arginase, nor was it able to change the ability of IFNg and LPS to induce iNOS or IL-4 to induce arginase [42]. Thus, like Gas6, treatment of macrophages with TGFβ1 resulted in altered macrophage cytokine responses without changing expression of the prototypical effector molecules of M1 or M2 differentiated cells. The presence of a specific TGFβR inhibitor was able to inhibit the conversion to IL-10 production by irradiated cancer cells (Figure 4c); however, the TGFβR inhibitor was not able to restore TNFα production by macrophages (Figure 4c). To test the combination with Mertk inhibition, we co-cultured irradiated cancer cells with macrophages in the presence of a TGFβR inhibitor, a Mertk-Fc blocking antibody or the combination. We demonstrated that irradiated cancer cells redirect macrophages to secrete suppressive cytokines, and both Mertk-Fc and TGFβR inhibitor partially block suppressive cytokine secretion (Figure 4d), but that the combination of the TGFβR inhibitor together with a blocking MertkFc fusion protein was able to completely inhibit the co-culture induced switch to IL-10 production and importantly was able to restore TNFα production in response to LPS stimulation (Figure 4d). These data demonstrate that Mertk ligation and TGFβ each individually prevent proinflammatory differentiation of macrophages, and combined blockade permits proinflammatory differentiation even in the presence of dying cancer cells.

**Figure 5:**
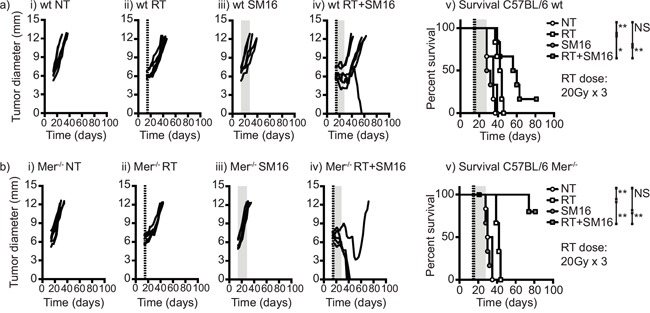
The combination of Mertk knockout and TGFβ inhibition permits tumor cure following RT of poorly immunogenic tumors **a.** C57BL/6 wild-type or **b.** C57BL/6 Mertk^−/−^ mice were challenged with Panc02 pancreatic adenocarcinoma and tumors were left untreated or treated on d14 with 20Gy x3 of focal radiation to the tumor (dashed lines). Mice were additionally treated with control food or food containing the orally bioavailable TGFβ inhibitor SM16 (shading). Graphs show tumor size in individual mice: i) untreated; ii) RT alone; iii) SM16 alone; iv) RT+SM16; v) Overall survival. Results are representative of two or more experimental repeats of 6-8 mice per group. Key: *=p<0.05; **=p<0.01; ***=p<0.001; NS = not significant.

In view of these data, we tested the effect of loss of Mertk and TGFβ signaling on radiation therapy of Panc02 tumors *in vivo*. To block TGFβ *in vivo* we treated wild type or Mertk knockout mice with the orally bioavailable small molecule TGFβR1 inhibitor SM16 [42] for two weeks following treatment with radiation therapy (Figure 5). As before, tumor growth and therapy were identical in wild-type and Mertk^−/−^ mice (Figure 5) and as we have previously shown, TGFβR inhibition alone did not significantly alter tumor growth [42]. When combined with radiation therapy, TGFβR inhibition extended survival in wild-type mice but in Mertk^−/−^ mice TGFβR inhibition was dramatically more effective and resulted in tumor cures (Figure 5b). Importantly, this combination of Mertk^−/−^ and TGFβR inhibition did not affect tumor growth unless radiation therapy was present, suggesting that the large-scale cell death induced by radiation therapy was required to initiate this response. During tumor rejection, Mertk^−/−^ mice treated with TGFβR inhibitors frequently exhibited either moist or dry desquamation in the radiation field that was not seen to any significant degree in any other group. This increased toxicity of radiation therapy resolved over time and resulted in a scarred treatment site but no other detectable problems in survivor mice. These data demonstrate that radiation therapy in the presence of combined loss of Mertk and TGFβR signaling is curative even in a highly unresponsive pancreatic adenocarcinoma, and demonstrates that therapeutically manipulating the macrophage response to dying cells in the tumor environment is a potential strategy to enhance the efficacy of radiation therapy.

## DISCUSSION

Tumor-associated macrophages are linked to poor prognosis in cancer patients, and represent a tempting therapeutic target. In the field of tumor immunotherapy in particular, there is generally an interrelationship between tumor macrophages and T cells, such that tumor macrophages can potently suppress T cell targeted immunotherapies [15, 45, 46]. This suppression of T cells by macrophages may be a critical component of inflammatory resolution and wound healing under normal conditions, but is also triggered by damage to the tumor environment. Our studies thus far indicate that the macrophage response to cancer cell death following cytotoxic therapy results in a shift to wound healing in the tumor environment that permits recurrence and outgrowth of residual cancer cells [18, 47]. Other groups have shown that decreasing the number of tumor macrophages by targeting CSF1R has proven to be an effective combination with both chemotherapy and radiation therapy [15, 16, 35, 37, 46]. Those macrophages that remain following CSF1R therapy are less suppressive [46]; however, it is noticeable that this therapeutic combination does not provide cures [15, 16, 35, 37, 46]. We hypothesize that while depletion only removes the negative signal, repolarizing macrophages to a pro-inflammatory state provides an additional positive signal that supports tumor clearance.

The Mertk receptor represents a very interesting therapeutic target both due to its highly specific expression on macrophages, and its role in the response to dying cells. Similar findings have been reported for MFG-E8, which also participates in the phagocytosis of apoptotic cells. Administration of MFG-E8 to macrophages suppresses proinflammatory responses [48, 49], and MFG-E8 blocking antibodies while ineffective as single agents result in tumor control when combined with chemotherapy or radiation therapy [50]. Since MFG-E8 signaling occurs in part through Mertk [32], it is possible that Mertk knockout mice lose the effects of both MFG-E8 and Gas6 mediated recognition of apoptotic cells. In some circumstances, Mertk is ectopically expressed on cancer cells but not on normal cells [51, 52], making it an additionally relevant target. Small molecule drugs targeting Mertk and the TAM family member Axl are in development for patients with acute myeloid leukemia and acute lymphoblastic leukemia [53]. Based on the data presented here, we would propose that optimal clinical translation of these agents for patients with solid tumors including colorectal carcinoma and pancreatic adenocarcinoma would involve radiation therapy delivered during Mertk blockade. In addition, a novel class of therapeutic antibodies has been developed that blocks the recognition of dying cells by phagocytes [54]. These antibodies that bind to exposed phosphatidylserine (PS) either directly or via PS-binding proteins, have been shown to significantly enhance the efficacy of radiation therapy [55] and chemotherapy [56], and act to redirect suppressive macrophage polarization [57]. The PS-targeting antibody Bavituximab is in late phase clinical trials in combination with cytotoxic therapy. However, as we have demonstrated, in some settings Mertk blockade is not sufficient to restore inflammatory functions in macrophages and is not sufficient for efficacy. We demonstrate that small molecule inhibition of TGFβR is a dramatically effective partner when combined with loss of Mertk signaling and would be an interesting combination for further development. Our prior studies demonstrated that the combination of small molecule inhibition of TGFβR with radiation therapy in responsive tumors resulted in a reduction in T regulatory cells in the tumor and efficacy was entirely dependent on CD8 T cells [42]; thus, it is possible that the *in vivo* function of TGFβ inhibition includes additional mechanisms in addition to, or instead of changing the macrophage response to dying cells. This is an area of ongoing study.

Mertk ligation has been shown to activate the MEK-ERK-Tpl2- NFκB p105 complex, and Mertk ligation has been shown to block conventional p50:p65 NFκB signaling [58]. Our data also demonstrates that NFκB p50 accumulation may be a mechanism of action of Mertk ligation and confirms both Mertk and NFκB1 p50 as potential target genes to improve tumor control to radiation therapy. It is particularly interesting that Mertk ligation changes the response of macrophages to stimuli without causing M1 or M2 polarization, via upregulation of NFκB p50. However, high NFκB p50 expression is also a feature of M2-polarized tumor macrophages [59] and LPS tolerized macrophages [60]. It is possible that in these circumstances ligation of Mertk by Gas6 on apoptotic cells may provide little additional signal to these previously polarized macrophages, but this remains to be determined. In view of these data, it is important to note that since subtle changes in the proportion of NFκB signaling molecules can alter the response of otherwise identical macrophages this may limit our ability to predict macrophage responses to stimuli based on surface phenotyping alone.

Though the responses are compelling, toxicity is increased where both Mertk and TGFβR signaling are blocked. In our model, close targeting of the tumor using CT-guided therapy limited the consequences to the animal; however it is possible that this strategy is too toxic for treatment plans with significant doses to critical structures. In our studies we are using high hypofractionated doses to model SBRT based on our combination immunotherapy studies in stage IV melanoma patients [61]. One distinction between our studies and patients treated at these doses is that while patients have *local* response rates of over 90% to metastases in the lung or liver [61, 62], in the mouse we see consistent local failure with radiation alone. In the setting of effective local control in patients it is likely most important to leverage locally generated immune responses to affect distant, untreated tumors and microscopic disease which is an extremely rare event in the absence of systemic therapy [63]. We believe that short course radiation represents a superior partner for immunotherapies to avoid repeated kill of lymphocytes [64] and provides acute rather than chronic antigen release. However, we also suspect that high, hypofractionated doses engender a stronger tissue repair response that limits the adaptive immune response. It remains to be determined whether changing the dose and fractionation of radiation changes the efficacy of loss of Mertk combined with TGFβ blockade and whether it changes the toxicity to normal tissue. This is an area of ongoing study. Similarly, it remains to be seen if other cytotoxic therapies such as chemotherapy will show enhanced efficacy when combined with loss of Mertk. Despite tumor cure in Mertk^−/−^ mice, in the days immediately prior to cure we do not see evidence of increased T cell infiltration into the tumor compared to wild-type mice (data not shown). We believe this is due to loss of Mertk resulting in decreased T cell suppression in the tumor environment rather than increased numbers of T cells, but the contribution of adaptive immune cells remains to be determined, including potential effects on distant, untreated tumors.

The data presented here show the dramatic difference between the fact of radiation-induced cancer cell death and the response of the host to cell death. Irradiated cancer cells are known to display a small-scale early apoptotic response to radiation therapy, but then the remaining cells die a slower clonogenic death through mitotic catastrophe. Thus, radiation results in a chronic exposure to dying cells. It is possible that since phagocytosis of dying cells is impacted, dying cancer cells will proceed to secondary necrosis with associated release of proinflammatory adjuvants. We and others have previously demonstrated that macrophages are critical sensors of apoptotic versus necrotic cells and engineering non-apoptotic cell death of cancer cells significantly improves anti-tumor immune control of tumors [3, 5, 65]. It is possible that strategies that increase levels of cell death in response to cytotoxic therapies will have only incremental increases in efficacy unless they reach the point where all cells are killed, whereas our data shows that changing the host response to cell death has the potential to dramatically change outcomes without changing the number of dying cells.

## MATERIALS AND METHODS

### Ethics

All animal protocols were approved by the Earle A. Chiles Research Institute IACUC (Animal Welfare Assurance No. A3913-01).

### Animals and cell lines

The CT26 murine colorectal carcinoma [66] (BALB/c), the 4T1 mammary carcinoma cell line [67] (BALB/c), the B16 melanoma (C57BL/6), Raw264.7 monocyte/macrophage cell line [68] (C57BL/6), and the 3LL lung adenocarcinoma [69] (C57BL/6) were obtained from the ATCC (Manassas, VA). The Panc02 murine pancreatic adenocarcinoma cell line [70] (C57BL/6) was kindly provided by Dr. Woo (Mount Sinai School of Medicine, NY). Six-8 week old C57BL/6 mice and BALB/c were obtained from Charles River Laboratories (Wilmington, MA) for use in these experiments. Mice lacking Mertk (Mertk^−/−^) [29] were obtained from The Jackson Laboratories (Bar Harbor, Maine) and backcrossed greater than 8x to C57BL/6 or BALB/c mice using genotyping from Transnetyx (Cordova, TN) and selection of breeder mice with the optimum genetic background using speed congenic services from Dartmouse (Lebanon, NH).

### Reagents

The orally bioavailable small molecule inhibitor of TGFβ, SM16, was obtained under a material transfer agreement from Biogen Idec (Cambridge, MA) and was incorporated into standard Purina rodent chow (#5001) by Research Diets (New Brunswick, NJ) at a concentration of 0.3 g SM16 per kg chow (0.03%) as previously described [71]. A calorie and nutrient-matched diet without SM16 (Purina) was used as the control diet. The Mertk-Fc fusion protein and recombinant Gas6 were obtained from R&D systems (Minneapolis, MN). Recombinant TGFβ1, IFNγ and IL-4 were obtained from Ebioscience (San Diego, CA). Ultrapure LPS was obtained from InVivogen (San Diego, CA).

### Gene expression analysis

The results of gene expression analysis of tumor-infiltrating macrophages has previously been published [18] and gene expression data has been uploaded to GEO (Accession number GSE34206). Data was analyzed using GeneSifter (Geospiza Inc, Seattle, WA) to generate normalized gene expression profiles of each treatment condition and used to identify phagocytosis-related genes with significant changes between treatment groups. Analysis of pathway interactions was performed using Cytoscape [72]. Analysis of gene expression in immune cell populations was performed on data generated by The Immunological Genome Project Consortium [73] and analyzed using tools provided at www.immgen.org.

### *Ex vivo* macrophage treatments

To generate bone marrow-derived macrophages, bone marrow cells isolated from long-bones of wild-type or Mertk^−/−^ mice were cultured for a total of 7 days in complete media containing 40ng/ml MCSF (Ebioscience), with additional growth medium provided after 3 days of culture. Adherent cells were harvested and macrophage differentiation confirmed by flow cytometry for CD11b, F4/80, Gr1 and IA. Macrophages were differentiated into M1 or M2 phenotypes by culturing for 24 hours in the presence of 10ng/ml IFNγ + 1μg/ml LPS or 10ng/ml IL-4, respectively as previously described [47], or treated with 500μg/ml recombinant Gas6. For co-culture, Panc02 or 4T1 cells were irradiated with 10Gy of radiation using a cesium source, and 1 × 10^4^ cancer cells were co-cultured with 1 × 10^4^ Raw264.7 cells or 2 × 10^4^ primary bone marrow macrophages in the presence or absence of TGFβ1, Mertk-Fc or Gas6 in replicate wells of 96-well u-bottom plates for 24 hours before treatment with 100ng/ml LPS. Supernatants were collected after a further 48 hours and tested for cytokine levels by ELISA using matched antibody pairs specific for TNFα and IL-10 (R&D Systems, Minneapolis, MN) against a standard curve of recombinant cytokine or using custom cytokine bead assays (Life Technologies, Grand Island, NY) run on a Luminex 100 array reader.

### Western blotting

Cells were lysed in RIPA buffer and denatured in SDS loading buffer containing beta 2-mercaptoethanol, electrophoresed on 10% SDS-PAGE gels and transferred to nitrocellulose. Blocked blots were probed overnight at 4°C with primary antibodies followed by HRP-conjugated secondary antibodies. Binding was detected using a Pierce SuperSignal Pico Chemiluminescent Substrate (Thermo Fisher Scientific, Rockford, IL) and exposure to film.

### CT-guided radiation therapy of tumors in mice

Tumors were inoculated s.c. in the right flank and allowed to establish for 10–14 days before initiation of treatment. Radiation was delivered using the Small Animal Radiation Research Platform (SARRP, XStrahl, Gulmay Medical, Suwanee, GA). Using a cone-beam CT scan with 360 projections, the tumor was visualized, the isocenter was placed within the tumor and a 10 × 10mm or 5 × 5mm collimator was utilized to deliver radiation. Dosing was based on recent clinical studies [61], with one to three daily 20Gy treatment fractions. Where specified, SM16 was present in the diet for two weeks following radiation therapy before return to normal diet in all groups.

### Cytokine bead assay

Tumors were harvested on ice and homogenized in 4.5μl PBS containing 1x HALT protease inhibitor (Thermo Fisher Scientific) per mg tissue. The cell debris was removed by centrifugation at 14000 x g for 15 minutes at 4°C, and supernatants were stored in aliquots at −80°C until used. Cytokine levels in the supernatants were detected using a murine multiplex bead assay (Life Technologies, Grand Island, NY) and read on a Luminex 100 array reader. Cytokine concentrations for replicates of each tumor sample were calculated according to a standard curve.

### Statistics

Data were analyzed and graphed using Prism (GraphPad Software, La Jolla, CA). Individual data sets were compared using Student's t-test and analysis across multiple groups was performed using ANOVA with individual groups assessed using Tukey's comparison. Kaplan Meier survival curves were compared using a log-rank test.
